# A distance-field based automatic neuron tracing method

**DOI:** 10.1186/1471-2105-14-93

**Published:** 2013-03-12

**Authors:** Jinzhu Yang, Paloma T Gonzalez-Bellido, Hanchuan Peng

**Affiliations:** 1 Current address: Key Laboratory of Medical Image Computing, Ministry of Education, Northeastern University, Shenyang, China; 2 Janelia Farm Research Campus, Howard Hughes Medical Institute, Ashburn, VA, USA; 3 Current address: Marine Biological Laboratory, Woods Hole, MA, USA; 4 Current address: Allen Institute for Brain Science, Seattle, WA, USA

## Abstract

**Background:**

Automatic 3D digital reconstruction (tracing) of neurons embedded in noisy microscopic images is challenging, especially when the cell morphology is complex.

**Results:**

We have developed a novel approach, named DF-Tracing, to tackle this challenge. This method first extracts the neurite signal (foreground) from a noisy image by using anisotropic filtering and automated thresholding. Then, DF-Tracing executes a coupled distance-field (DF) algorithm on the extracted foreground neurite signal and reconstructs the neuron morphology automatically. Two distance-transform based “force” fields are used: one for “pressure”, which is the distance transform field of foreground pixels (voxels) to the background, and another for “thrust”, which is the distance transform field of the foreground pixels to an automatically determined seed point. The coupling of these two force fields can “push” a “rolling ball” quickly along the skeleton of a neuron, reconstructing the 3D cell morphology.

**Conclusion:**

We have used DF-Tracing to reconstruct the intricate neuron structures found in noisy image stacks, obtained with 3D laser microscopy, of dragonfly thoracic ganglia. Compared to several previous methods, DF-Tracing produces better reconstructions.

## Background

In neuroscience it is important to accurately trace, or reconstruct, a neuron’s 3D morphology. The current neuron tracing methods can be described, according to the necessary manual input, as being manual, semi-automatic or fully automatic. Neurolucida (MBF Bioscience), a largely manual technique, uses straight line-segments to connect manually determined neuron skeleton locations drawn from the 2D cross-sectional views of a 3D image stack. In contrast, semi-automatic methods need some prior information, such as the termini of a neuron, for the automated process to find the neuron skeleton. For example, the semi-automatic Vaa3D-Neuron 1.0 system (previously called “V3D-Neuron”) [1,2] has been used in systematical and large-scale reconstructions of single neurons/neurite-tracts from mouse and fruitfly [3,4].

However, for very complicated neuron structures and/or massive amounts of image data, the semi-automatic methods are still time-consuming. Thus, a fully automated tracing method is currently highly desired. Early fully automated methods used image thinning to extract skeletons from binary images [5-7].

These methods iteratively remove voxels from the segmented foregroun region surface of an image. In addition, neuron-tracing approaches based on pattern recognition were also developed ([8-13]). However, in cases of low image quality, the tracing accuracy may be greatly compromised. The model-based approaches, such as those that use a 3D line, sphere or cylinder for identifying and tracing the morphological structures of neurons, are relatively more successful ([14-17]). These methods can also be guided using both global prior information and local salient image features ([2,18,19]). While the basis of most existing methods is to grow a neuron structure from a predefined or automatically selected “seed” location, the all-path pruning method [20] that iteratively removes the redundant structural elements was recently proposed as a powerful alternative.

**Figure 1:**
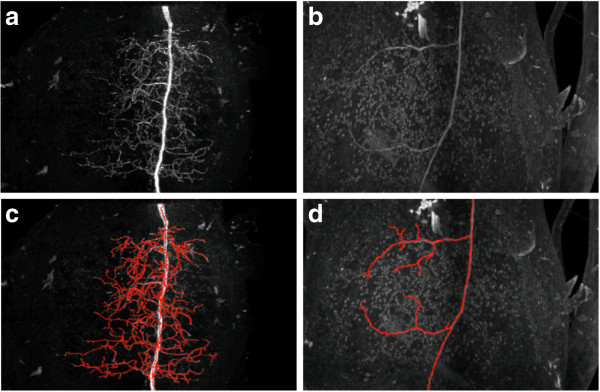
**Examples of 3D confocal images containing complicated dragonfly neurons and heavy noise.** (**a**) A dragonfly neuron with highly complex structures. (**b**) Noise-contaminated image. (**c**) (**d**) DF-Tracing reconstructions (red color, only skeletons are shown) of (**a**) and (**b**), respectively.

Despite such a large number of proposed neuron tracing algorithms ([14,21]), few can automatically trace complicated neuron structures set in noise-contaminated microscopic images (Figure 1 (a) and (b)). Here we report a new method, named DF-Tracing (‘DF’ for “Distance Field”), which meets this challenge. We tested DF-Tracing with very elaborate images of dragonfly neurons. Without any human intervention, DF-Tracing produced a good reconstruction (Figure 1 (c) and (d)), comparable in quality to that of human manual work.

## Method

**Figure 2:**
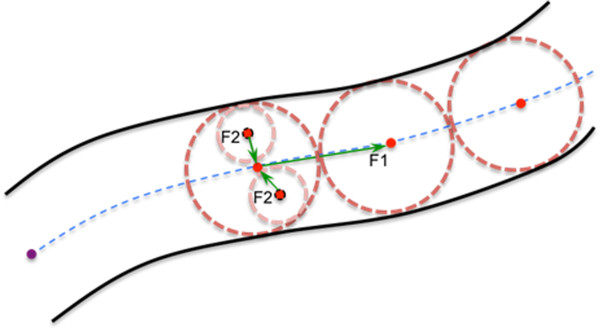
**Schematic view of a neuron segment.** Circles/spheres: reconstruction nodes, of which their centers (red dots) indicate the skeleton (blue curve) of this segment. Each reconstruction node has its own cross-sectional diameter estimated based on image content. *F*_1_ and *F*_2_ indicate the thrust and pressure forces of our neuron tracing method.

A reconstructed neuron (e.g. Figure 1 (c) and (d)) has a tree-like structure and can be viewed as the aggregation of one or more neurite segments. Each segment is a curvilinear structure similar to Figure 2. When a neuron has multiple segments, they are joined at branching points. The neuron structure can thus be described with a SWC format [22], where there are a number of reconstruction nodes and edges. Each node stands for a 3D spatial location (x,y,z) on the neuron’s skeleton. Each edge links a node to its parent (when a node has no parent, then its parent is flagged as -1). The cross-sectional diameter of the neuron at the location of each node is also calculated and included in SWC format. Therefore, to produce a neuron reconstruction, two key components are (a) determination of the skeleton, i.e. ordered sequence of reconstruction nodes, of this neuron, and (b) estimation of the diameters at each node’s location.

An intuitive way to trace a neuron is to start from a predefined or automatically computed location, called “seed”, to grow the neuron morphology until it covers all visible signals in the image. If the image foreground region that corresponds to a neuron can be well extracted, the problem reduces to determine the complete set of reconstruction nodes, i.e. skeletonize the neuron, using the foreground mask of the image, followed by estimating the cross-sectional diameter of all reconstruction nodes.

We follow this intuition and design the following three-stage neuron-tracing algorithm:

(Step 1) Enhance line-like structures in the image, followed by adaptive thresholding to remove non-neuron background and noise. (2.1)

(Step 2) Skeletonize the neuron using coupled distance fields. (2.2)

(Step 3) Assemble multiple spatially disconnected pieces of the traced neuron into the final result. (2.3)

### Preprocessing: extraction of neuron signal

Since a neuron segment looks like a line or a tube (Figure 2), anisotropic filtering of image noise can enhance the neuron signal in an image. We use nonlinear anisotropic filtering for signal enhancement, followed by automated thresholding.

The key idea of anisotropic filtering is to calculate image features that signify the orientation preference of local image areas. We follow the classic Hessian matrix based method, which detects the curvilinear structures in images [23]. Let *u*(*x,y,z*) stand for an image, where *x, y, z* are the spatial coordinates. We use ∇*u* to denote the image intensity gradient. A filtered image pixel will take the following value *v*,

(1) $$v=\exp\left(-{\left(\left|\nabla u\right|\right)}^{2}\right)f\left(u\right).$$

The function *f*(*u*) is defined using the Hessian matrix of each image pixel *u,*$H=\left[\begin{array}{ccc} u_{\mathrm{xx}} & u_{\mathrm{xy}} & u_{\mathrm{xz}}\\ u_{\mathrm{yx}} & u_{\mathrm{yy}} & u_{\mathrm{yz}}\\ u_{\mathrm{zx}} & u_{\mathrm{zy}} & u_{\mathrm{zz}} \end{array}\right],$ where ***H*** is indeed symmetric. Of note, the Hessian method has been well used in medical image computing, especially vessel enhancement and segmentation ([24,25]).

To do so, we compute the eigenvalues of *H*, denoted as *λ*_1_, *λ*_2_, and *λ*_3_ (*λ*_1_ ≥ *λ*_2_ ≥ *λ*_3_). Interestingly, when the brighter pixel intensity indicates the stronger signal and a line-look structure is present at the current pixel location, there is *λ*_1_ ≈ 0 and *λ*_1_ >> {*λ*_2_, *λ*_3_} [23]. Therefore, we explicitly detect if such conditions will be met for each pixel, and define the following function *f*(*u*).

(2) $$f\left(u\right)=\{\begin{array}{l} \sum_{i}^{3}=1\;a_{i}\lambda_{i}k_{i\;}\;\left(\lambda_{1}\approx 0,\lambda_{1}\gg \lambda_{2},\lambda_{1}\gg \lambda_{3}\right)\\ \;0\;\left(\mathrm{otherwise}\right) \end{array}$$

where α_*i*_ (*i* = 1, 2, 3) are pre-defined coefficients (α_1_=0.5, α_2_=0.5, α_3_=25), $k_{i}=\text{exp}\left(-\lambda_{i}^{2}/\sum_{i}^{3}{=}_{1}\lambda_{i}^{2}\right)$.

After signal enhancement, extracting the neurite foreground using global thresholding is straightforward. We determine the threshold with the following iterative process. First, the average image intensity is taken as a candidate threshold. We use this candidate threshold to divide the image into two portions: pixels with higher, or lower intensity. We then calculate the average of the two mean intensity values of these two portions and use it as a new candidate threshold. This process is iterated until the candidate threshold value no longer changes. This converging threshold is used as the final global threshold: any image pixel with intensity higher than this threshold is part of the so called “image foreground”, which is assumed to contain the neuron signal.

Lastly, since the 3D-extracted image foreground could contain multiple neurite areas and noise, neuron reconstruction is carried out in individual areas, which are “stitched” together via post-processing. Discarding the very small pieces (smaller than 10 pixels) removes the noise still present in the image foreground.

### Neuron tracing using coupled distance fields

Figure 2 shows that the skeleton of a neuron segment is essentially the medial axis of this segment. How can the medial axis be recovered? Possibly the simplest way is image thinning, which unfortunately has two well-known problems: (a) sensitivity to orientation of the image region of interest, and (b) unnecessary forks of the skeleton at the ends of the image region. Another intuitive approach is to use tube fitting (e.g. [17]) or a rolling ball fitting. Below, we present a simple method that works robustly without a parameter.

Distance transform of an image region *R* with respect to another image region *T* is defined as for each image pixel in *R*, replace its intensity using the shortest distance to *T*. Typically *T* is selected as the image background, but it can also be selected as any specific image pixel. We have the following observation of Figure 2.

• In the distance transform of a neuron segment with respect to an arbitrarily selected seed location, *s*, at a tip point of the neuron segment, the distance-transformed pixel intensity has a larger value than those of the nearby foreground pixels. In another word, any tip/terminal point in a neuron structure will have a local maximum on the boundary pixels in this distance field.

• In the distance transform of a neuron segment to the image background, the boundary pixels will have value 1 and the skeleton points will form a ridge of local maximal values, compared to all other image pixel locations that are orthogonal to the tangent direction of the skeleton. Extraction of the ridge will skeletonize the neuron segment.

• Assume we have a rolling ball that is pushed forward by these two coupled distance “force” fields. It can be seen that this ball will move toward the skeleton from any starting location and then along the ridge curve. Following this trajectory we can extract the neuron skeleton quickly. For this reason, in Figure 2 we call these two force fields “thrust” (for *F*_1_, which is the distance transform field of the foreground pixels to an automatically determined seed point) and “pressure” (for *F*_2_, which is the distance transform field of foreground pixels (voxels) to the background) fields, respectively.

• Multiple traced neuron segments can be merged at their convergence point to reconstruct the tree-like structure of a neuron.

Based on these observations, we have designed the following DF-Tracing algorithm:

(1) Detect neuron region(s) in 3D using method in 2.1. Multiple spatially non-connected neuron regions may be produced.

(2) For each neuron region *R*, find the set of boundary pixels *B*, which is defined the set of pixels having at least one neighboring pixel (26-neighbors in 3D) as the background.

(3) Arbitrarily select a seed location, *s*, from the boundary pixel set *B*.

(4) Compute both the thrust and pressure distance fields with respect to *s* and *B*, using the entire image.

(5) In the thrust field, detect the set of all local maxima locations, M.

(6) For each point *t* ∈ *M*, set an initially empty skeleton *C*(*t*) to {*t*}. Denote the last added skeleton point as *p* (initially *p* = *t*).

(7) Denote π(*p*) = *R* ∩ Ω(*p*) (Ω is the 26-neighborhood of a pixel in 3D). For all pixels *q* ∈ π(*p*), find q* that has the largest pressure field value.

(8) If q* also has a lower thrust field value (note that now we are back-tracing a path) than *p*, then add q* to the skeleton set.

(9) Assign q* to *p*, and repeat steps 6 and 7 until the seed location s is met. This completes the skeleton of a neuron segment *C*(*t*).

(10) Merge the common portion of multiple skeletons.

(11) Use the pressure field value of skeleton points as the respective values for the radius estimation.

(12) Assemble multiple neurite reconstructions for all neuron regions using the post-processing method in 2.3.

In its implementation, our DF-Tracing algorithm can be further optimized. Indeed, steps 6-9 can be parallelized. Instead of finding the complete skeleton for each terminal point *t* ∈ *M* sequentially, we can grow every skeleton one step at a time in parallel. A skeleton stops growing when either the seed location or any other skeleton pixel location has been reached. This process iterates until all skeletons stop growing. The parallelized algorithm will also avoid step 9, i.e. merging common portions of skeletons. In addition, to save computational time when calculating the distance fields, we use the city block distance [26] instead of the Euclidean distance.

### Post-processing: produce the complete reconstruction

The neuron-tracing algorithm in 2.2 can return a tree-like structure for a single 3D connected neuron region. In case that the neuron foreground extraction (2.1) produces multiple spatial disconnected neuron regions, we will have multiple neuron trees. Often these pieces need to be assembled into a full reconstruction. Since a gap between two disconnected neuron regions is typically small; only the nearby pieces with a separation smaller than two times the radius of the nearest nodes are connected. Then, an arbitrarily selected “root” location (usually the first leaf node in the SWC representation) is used to sort the order (i.e. parent-children relationship) of all neuron reconstruction nodes. Finally, pruning the very short branches whose lengths are less than two pixels completes the reconstruction.

### Pros and cons of DF-tracing, and comparison to other methods

DF-Tracing is an efficient, deterministic, and essentially parameter-free method (for the core part of coupled distance fields). Compared to many previous neuron-tracing methods, this new method avoids the complication introduced by the previous need to select parameters. In addition, DF-Tracing is a local search method, similar to the major body of existing neuron-tracing techniques. While local search cannot guarantee the global correctness of the final reconstruction, compared to those use the global guiding information (e.g. Vaa3D-Neuron 1.0), DF-Tracing has the advantage that it uses a smaller amount of computer memory. Moreover, we note that the termini produced in the thrust field (step 5 in the DF-Tracing algorithm) could be used as global guiding prior information for the Vaa3D-Neuron system. In that sense, the steps 6-10 in DF-Tracing is basically equivalent to the shortest path algorithm in the graph-augmented deformable model [2]. However, DF-Tracing does not need to literally produce the graph of image pixels and thus uses less computer memory.

DF-Tracing has several caveats that deserve further improvement. First, it is based on distance transform, which may be sensitive to neuron boundary and also anisotropic neuron structure in 3D images. This can be refined in the future by (1) replacing distance transform with a more robust statistical test method, similar to the diameter estimation method used in Vaa3D-Neuron and (2) smoothing the contour/edge of the extracted image foreground to make the distance transform more robust to image noise. Second, the post-processing of DF-Tracing can be further improved by adding machine learning methods (e.g. [27]). Third, the preprocessing step can be further improved using a multi-scale anisotropic filtering approach [23].

## Results and discussion

To assess the DF-Tracing method, we consider several datasets, especially one consisting of 3D confocal images of dragonfly neurons (species *L. luctuosa*), which have very complicated neuron arborization patterns, heavy noise and uneven image background (Figure 1 (a) and (b)). We also tested DF-Tracing using neuron images from other organisms, such as the fruit fly (species *D. melanogaster*). We compared DF-Tracing with existing automatic approaches, especially NeuronStudio [28] and iTube [17], and the semi-automatic approach Vaa3D-Neuron 1.0 ([1]).

We tested the neuron-tracing algorithm on 3D confocal image stacks of neurons in dragonfly (data set was obtained from [29]) and *Drosophila* (data from the Digital Reconstruction of Axonal and Dentritic Morphology (DIADEM) competition (http://www.diademchallenge.org).

### Neuron signal enhancement and neuron tracing

**Figure 3:**
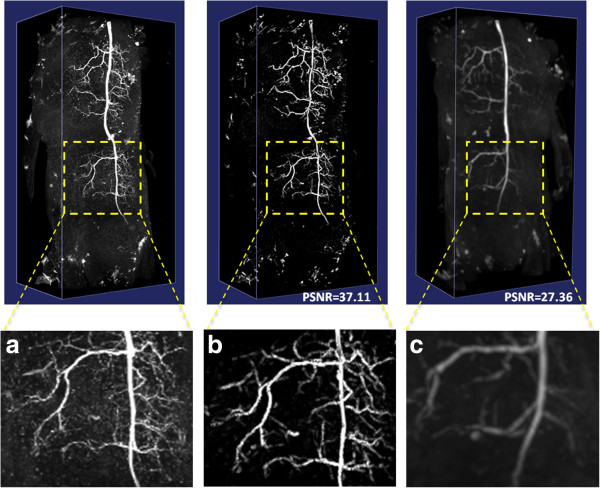
**Image denoising results using 3D confocal images containing dragonfly neurons.** (**a**) Original images. (**b**) Our anisotropic filtering result. (**c**) Gaussian filtering.

Our dragonfly image stacks are noisy and have low contrast (Figure 1(a) and (b), thus they are good test cases to examine the neuron signal enhancement method in 2.1. We added Poisson noise to the original image and compared the results to Gaussian-filter based denoising. As shown in Figure 3, our anisotropic filtering method is able to produce much better peak signal-to-noise ratio (PSNR) ([30]). Visually, our method also preserves and enhances the neurite signal significantly.

**Figure 4:**
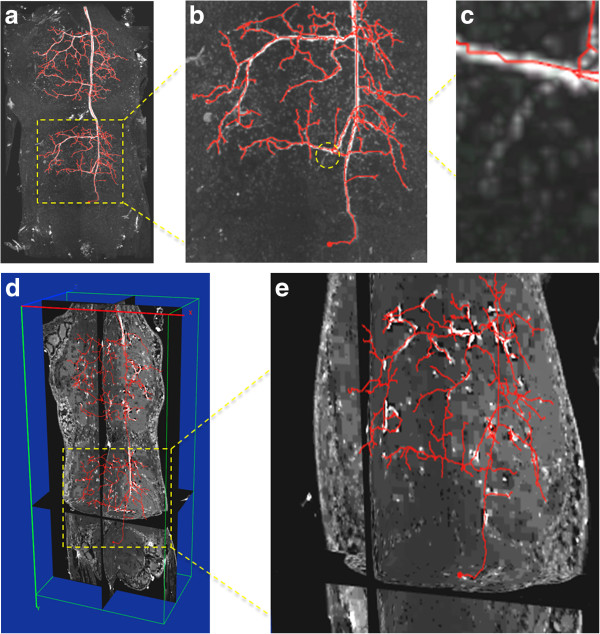
**Results from the DF-Tracing method using 3D confocal images containing dragonfly neurons.** (**a**) The 3D maximum intensity projection of the input data and the neuron tracing results (red). (**d**) The cross-sectional view (image intensity brightened for better visualization). (**b**) (**c**) and (**e**) the zoom-in the respective areas.

We then used DF-Tracing to trace the neuron in Figure 3 (a). After filtering, there are many disconnected neuron regions (Figure 3 (b)). DF-Tracing successfully traced all individual regions and merged the final result (Figure 4). The final tracing result faithfully replicates the original neuron morphology. A few small branches are missing (Figure 4 (b)), which are due to the low image quality in the respective image areas. In summary, Figure 4 demonstrates that both the signal enhancement and tracing modules in the DF-Tracing algorithm yield a high performance.

### Neuron tracing: comparison with other methods

We compared DF-Tracing to the following three neuron-tracing programs, which are publicly available and have been used to produce several recent significant results in neuroscience.

(1) Vaa3D-Neuron 1.0 semi-automatic tracing ([2]);

(2) NeuronStudio automatic tracing (0.9.92 version, http://research.mssm.edu/cnic/tools-ns.html);

(3) iTube automatic tracing [17];

To compare the key tracing modules of different methods, we used one confocal image of fruitfly olfactory projection neuron, which has a very high signal-to-noise ratio and is also used in several previous studies ([31]). We binarized this image for testing.

**Figure 5:**
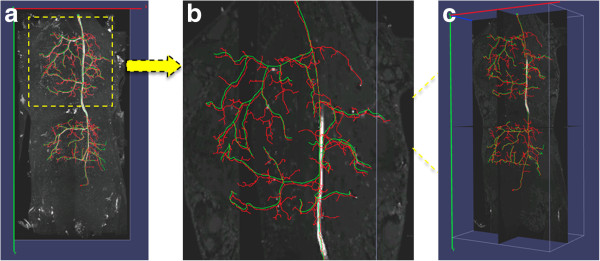
**Differences between DF-Tracing (red) and NeuronStudio (green) tracing methods using 3D confocal images containing dragonfly neurons.** (**a**) The 3D view. (**b**) The zoom-in the respective areas (**c**) The cross-sectional view. In all sub-figures, the skeletons are overlaid on top of the image. We intentionally offset these two reconstructions a little bit for clear visualization.

**Figure 6:**
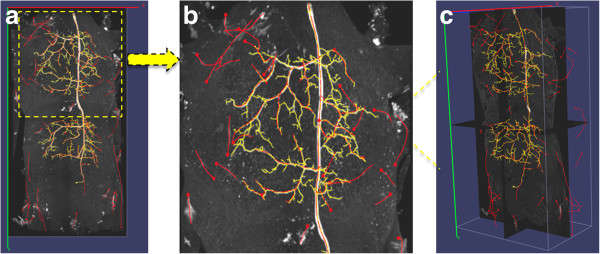
**Differences between DF-Tracing (yellow) and iTube (red) tracing methods using 3D confocal images containing dragonfly neurons.** (**a**) The 3D view. (**b**) The zoom-in the respective areas (**c**) The cross-sectional view.

We also compared the tracing performance of these methods for complicated neuron morphology using the dragonfly neurons. Due to the complexity of the neuron structure, it is very difficult to manually determine the end points of neurons within a day, thus it is impractical to directly use Vaa3D-Neuron 1.0 for these dragonfly neurons. Figures 5 and 6 show the tracing results produced by both NeuronStudio and iTube. Both methods missed a number of branches, whereas DF-Tracing reasonably recaptured the neurons’ morphology.

### Neuron tracing: quantitative analysis

**Figure 7:**
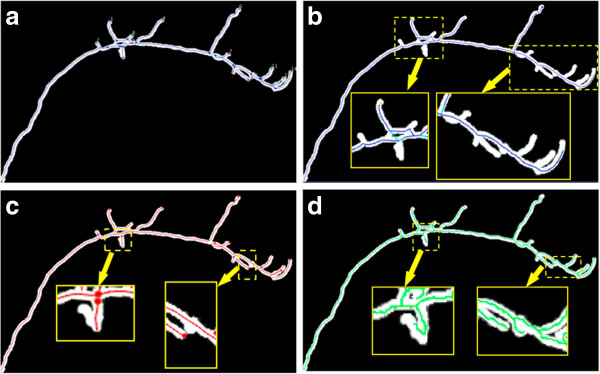
**Results of different tracing methods using a binary confocal image of a fruitfly olfactory projection neuron.** (**a**) V3D-Neuron 1.0. (**b**) NeuronStudio. (**c**) iTube. (**d**) DF-Tracing.

Figure 7 compares the results between tracing methods. With Vaa3D-Neuron, we manually selected all end-points of this neuron and inspected the results to produce the “ground truth” for evaluation (Figure 7 (a)). We selected a total of 17 points. It is apparent that the NeuronStudio result misses many branches (Figure 7 (b)). The iTube result includes most branches, but still misses a few, especially the highly curved structures (Figure 7 (c)). DF-Tracing produced the same result as the ground truth version (Figure 7 (d)). Table 1 summarizes the comparison quantitatively. It is clear that the DF-Tracing result is the best among these methods as all branches were correctly traced.

**Table 1:** Number of branches of different methods

**Method**	**V3D-Neuron 1.0**	**iTube**	**NeuronStudio**	**DF-Tracing**
Number of branches	17	15	9	17

**Figure 8:**
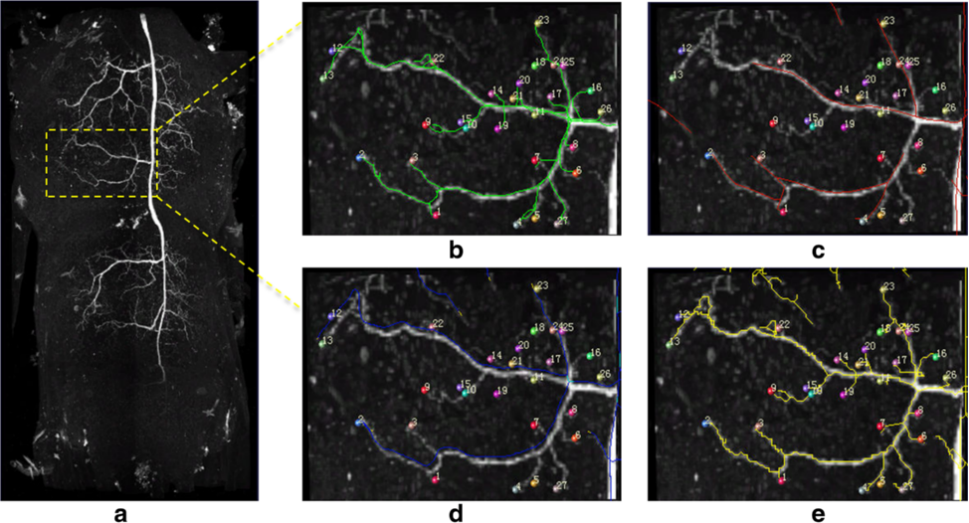
**Results of different tracing methods using a grayscale dragonfly confocal image.** (**a**) The 3D view. (**b**) Vaa3D-Neuron 1.0. (**c**) NeuronStudio. (**d**) iTube. (**e**) DF-Tracing.

We also compared the tracing results for a non-binary confocal image. We chose a local region of the dragonfly neuron (Figure 8 (a)). We also selected all end-point (totally 27) of this neuron and inspected the results to “ground truth” for evaluation (Figure 8 (b)). The NeuronStudio and iTube results miss many branches (Figure 8 (b) and (Figure 8 (c)). DF-Tracing produced the same result as the ground truth version (Figure 8 (d)). Table 2 summarizes the comparison quantitatively. There was also clear that the DF-Tracing result produced the best performance among these methods, as most branches were correctly traced.

**Table 2:** Number of branches of different methods

**Method**	**V3D-Neuron 1.0**	**iTube**	**NeuronStudio**	**DF-Tracing**
Number of branches	27	6	3	27

With regards to running time for tracing, Vaa3D-Neuron 1.0 is still faster than the new DF methods, although it requires some human-interaction time, with an execution time of around 10 seconds for the Figure 7 on an Intel Q6600 processor (2.40 GHz). The tracing time for Figure 8 is around 22 seconds. Table 3 summarizes the comparison time of different methods. It is clear that the time of DF-Tracing is currently the slowest of all automatic methods, but its accuracy is the best. However, it should be noted that the operations of DF-Tracing can be parallelized, and thus in a future implementation we hope to accelerate the speed by orders of magnitude through the use of multi-core processors and graphics processing units (GPUs).

**Table 3:** Tracing time of different methods

**Method**	**V3D-Neuron 1.0**	**iTube**	**NeuronStudio**	**DF-Tracing**
Tracing time of Figure 7	10 (s)	15 (s)	4 (s)	20 (s)
Tracing time of Figure 8	22 (s)	28 (s)	10 (s)	38 (s)

### Neuron tracing: robustness

**Figure 9:**
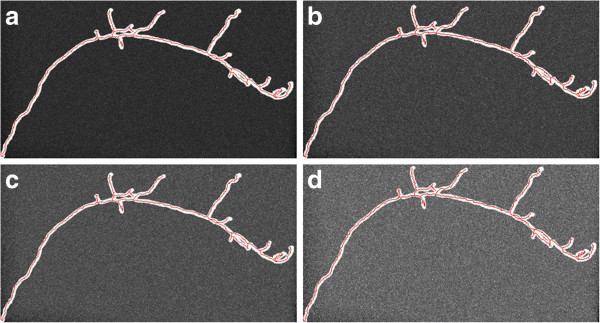
**Reconstructions produced for Gaussian-noise contaminated images.** (**a**) Gaussian noise variance v = 0.01. (**b**) v = 0.02. (**c**) v = 0.03. (**d**) v = 0.05.

We tested the robustness of DF-Tracing. We added Gaussian white noise of mean 0 and different variance *v* to the image in Figure 7(a), where *v* = 0.01, 0.02, 0.03 and 0.05 respectively. In this way, multiple reconstruction results were produced As shown in Figure 9. We computed the pair-wise spatial distance (SD) score of these reconstructions, as defined in [1]. The average SDs is 0.149 pixel, very close to 0.

**Figure 10:**
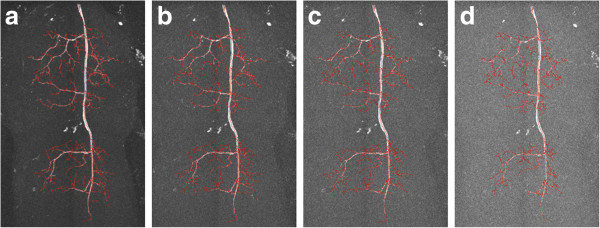
**Reconstructions produced for Gaussian-noise contaminated images.** (**a**) Gaussian noise variance v = 0.01. (**b**) v = 0.02. (**c**) v = 0.03. (**d**) v = 0.05.

We also tested the robustness of DF-Tracing for a non-binary confocal image. We added Gaussian white noise of mean 0 and different variance v to the image in Figure 8(a), where v = 0.01, 0.02, 0.03 and 0.05 respectively. In this way, multiple reconstruction results were produced. As shown in Figure 10, the test image contains various levels of noise. For example, when v = 0.05, most signals of the image have been contaminated, yet we can trace major neuron branching in the remaining visible image regions. We computed the pair-wise SD score of these reconstructions and the average of SDs is 0.62 pixel. These expe-riments demonstrate that our method can produce consistent and robust reconstructions.

### Automatic tracing of complicated morphology of many neurons

**Figure 11:**
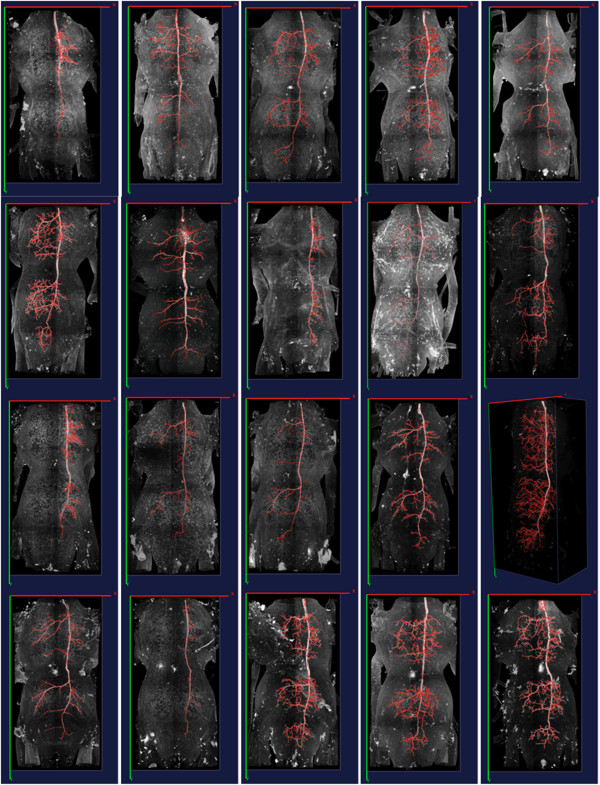
**Tracing results using 20 confocal 3D images containing different dragonfly neurons.** The reconstructed skeletons (red) are overlaid on top the maximum intensity projection of different confocal images for clear visualization.

In Figure 11, we tested the performance of DF-Tracing on 20 dragonfly neurons (thoracic ganglia) that have various levels of complexity and background noises. DF-Tracing reconstructed the morphology within a day on a MacBook Pro laptop. Fully manual tracing of same set of data would need at least tens of days. The biologist (PGB) in this study visually inspected all results and found all major neuron trunks and branches have been correctly traced. Of note, the complexity of morphology and high noise level make it very hard to produce faithful manual tracing, thus DF-Tracing is evidently a meaningful solution to this data set.

## Conclusion

We have developed a automatic neuron tracing method, DF-Tracing, that outperformed several previous automatic and semi-automatic methods in a very challenging set of dragonfly neurons with complex morphology and high noise levels. This method is efficient and essentially parameter-free. DF-Tracing has application potential in large-scale neuron reconstruction and anatomy projects.

## Competing interests

There are no competing interests.

## Authors’ contributions

HP conceived and supervised the study; JY designed algorithms; JY and HP wrote program and performed data analysis; PGB assisted in data analysis; all authors participated in the preparation of this manuscript. All authors read and approved the final manuscript.
